# Etiology and Diagnosis of Nymphomania

**Published:** 1887-10

**Authors:** C. H. F. Routh


### Selections.

### *ETIOLOGY AND DIAGNOSIS OF NYMPHOMANIA.*

By C. H. F. ROUTH, M. D.

Dr. Magnan divided sexual anomalies into four groups—the *purely spinal* cases; the *posterior cerebro-spinal;* the *anterior cerebro-*

*spinal,* and the *anterior cerebral,* or physical cases. [A translation of Dr. Magnan's paper can be found in the *Med. Abs.*, page 121, 1885.]

The writer mentions the case of a woman, aged seventy, the reverse of lustful, and yet defecation produced such sexual excitement that she was compelled to masturbate. She was cured by clitoridectomy. The second case was that of a high-born lady, thoroughly respectable in every way. The moment a part of the vagina behind the urethra was touched, the most violent excitement was induced. In a third case, there was a fibroid tumor and adherent left ovary, the same unnatural excitement followed the moment the ovary was touched.

As the author is considering the subject specially from a medico-legal point of view, his remarks chiefly concern the third class—anterior cerebro-spinal cases. In these cases, we can observe five characteristics: 1. There is a strong neurotic hereditary history. 2. They are, for the most part, agreeable, interesting, if not beautiful women, and have very special physical conformations. 3. They are intensely vain, and open to adulation. 4. There is generally some uterine or ovarian complication, and they are frequently masturbators. 5. They are indubitably the most decided liars in creation, yet they are under the firm delusion that they are speaking the truth all the while. The knowledge of these five peculiarities will often enable us to diagnose this pitiful condition, and both to remedy the disease and avoid any personal risk to ourselves.

These women are generally either very good-looking, or, at least, very interesting in their appearance; full of sympathy in their manner, having strong affections, and generally attractive to men because of their effeminate appearance. In social life, they are eminently agreeable, desirous to please, often the nicest young women in society; full of romance and fantastic longings. The pupils are generally much dilated, the eyes themselves exceedingly bright, furrowed and black under the eyelids, and they know well how to use them. When under the influence of any intoxicating drug, such as chloroform or inebriating drink, they very often, by their conversation before they go off completely, or the manner in which they conduct themselves upon the bed, betray the exalted state of their sexual feelings, and they will sometimes let out, while they are under this influence, sudden statements which will lead you to fear that they are anything but what they have appeared to be. Occasionally, on awakening out of this sleep, when they resume their faculties, they are under the impression that you have done something to them which was either immodest or immoral; and this is the more remarkable as they have all the while been surrounded by spectators, male and female, before whom any such imprudence could not have been committed. These are not necessarily bad women, impure or unchaste.

A girl about seventeen desired me to remove some needles from her body, which, she asserted, she had swallowed. She was very vain, thought herself extremely handsome, and that her form was quite that of a Venus. The first time she came to me I found three needles in her bosom, which I withdrew with some pain to herself. The next week the needles were lower down, about the umbilicus. Now there were four or five stuck in nearly the whole length, having just the head outwards. This led me to suppose that they must have been inserted from without; the third week she came to me with several needles in the lower part of the belly, about the pudenda. I at once came to the conclusion that these had been inserted by herself externally, with a view to display what she thought were personal attractions. On removing these, I cut her rather deeply, and I never saw her from that moment. This girl I had every reason to believe was a *virgo intacta*. One other case was that of a German lady—highly diseased, congestio uteri vaginitis, strong neurotic symptoms—in no way attractive as far as face, at least, was concerned, and, so far as I knew, thoroughly chaste. I found her in bed. To my surprise, she suddenly uncovered herself and I saw she was nude. I remonstrated and chided her. "Oh!" she said, "I only wished you to see how beautifully formed I was." This lady, a few months later, went stark mad, and is at present somewhat peculiar. It is extremely dangerous to treat such women harshly, but it is quite possible not to offend their vanity, and yet reprove them kindly.

The condition cannot always be made out in very young women; but past the age of twenty-six, if the habit has been continued for a long period of years, the symptoms of this habit will be very clear. First, the internal labia are extremely developed, projecting considerably beyond the external. Sometimes one internal labium projects extremely, generally of the side to which they are handled—and I have known a labium so long as to hang like the tongue of a bulldog.

When these internal lips are turned outwards on the under surface, an appearance very like psoriasis is seen—patches of bright shining scales, always dark, within the labium. It is sometimes, if a woman be very dark, nearly black. The clitoris is generally swollen and enlarged, or hard and small, and it cannot be pressed upon without making the woman jump, which is a symptom of its extreme sensibility. Inferiorly, the fourchette, instead of projecting as it does in young children and women, is hollowed out, as if concave, while the hairs for the most part have lost their curly character. There is, moreover, between the internal labia and clitoris, a white offensive secretion, not unlike that which would be produced by filling the crevices with white powder. When a vaginal examination is made, there is generally partial prolapse of the uterus, which may be within two or three inches of the external orifice. There may be or may not be redness of the organ, but there always is more or less leucorrhœa. I should remark here, if there be any syphilitic taint in the woman, there is much more hypertrophy apparent in these parts. These symptoms or signs may be present in other diseases of these parts, accompanied with great pruritus, as perhaps in some cases of diabetes and lupus, but I think as a whole they pretty accurately denote imprudent habits.

They are indubitably the most decided liars in creation, and yet they are under the firm delusion that they are speaking the truth all the while. This propensity seems to influence their whole nature. There is scarcely anything, never mind how absurd, which these women will not do or say to obtain sympathy or notoriety. It is also a curious phase in this disease, that like an epileptic or hysterical seizure, it seems to instigate other women to covet similar notoriety; and wheresoever a man is accused by one of these women, others will start up suddenly and make similar charges against the same man. This occurred, during the last few months, in two cases where medical men were cruelly accused.

The manner in which some women will simulate phantom tumors, by partial contraction of the abdominal parietes, is very remarkable. It shows their pertinacity in a purpose, for such contractions must be acquired after long practice. I had such a case, where the patient could put the liver against the abdomen so that any percussion gave full indication of a large tumor.

A young lady in a very good station of life charged three gentlemen with having ravished her. An examination found the clitoris very sensitive, and covered with a white secretion like moist fuller's earth; hymen intact, just admitting the little finger. This woman subsequently made overtures to some railway porters, and was placed in an asylum. A woman, past seventy, claimed to have become pregnant from some matter left on the seat of a w. c., where a man and woman had been together. A hospital nurse accused a house-surgeon of having ravished her while she was sleeping in the ward, her bed being between those of two other women. A married woman was in the habit of visiting medical men for pretended uterine disease. Her object was to tempt them to some indiscretion, with the purpose of accusing them to her husband.

The above cases will suffice to show the insane features of these hysterical cases. They bear a very close relation to those cases of nymphomania which are admitted to depend on actual disease of the brain. Let me refer to one or two of such instances.

The late Mr. North related the case of a lady, a great sufferer. He always visited her in the presence of her mother; but on one occasion, the mother being engaged, she begged him to go up and see her daughter. No sooner had he got into the bedroom, and she became conscious that the mother was not accompanying him, than she instantly exposed the whole of her person. He kindly rebuked her. She turned around to him and said, "Doctor, you do not know the agony which I endure every day from the passions that devour me." She was dead in three days.

Another instance was that of a charming lady who had lived in the family for fifteen years, and was much beloved. All her life-time she had been most devoted, a model of everything that was pure and holy. The poor girl became very ill, seized with marked hysterical mania. As there was a strong tendency to suicide, she was never left alone, but different members of the family used to watch by her bedside. At last the practitioner offered to sit up with her for one night. After all the family had gone to rest, she at once opened her mind to him, and urged him to have connection with her, which, of course, he refused. Three days after, that poor girl was a corpse.

It was but the other day that a married lady, also a model of everything that is pure and modest, told me that she sometimes suffered from such agony and excitement in the sexual organs, which lasted for not one hour, but four and five and six at a time, that if she were not kept back by strong religious feeling, she would have run into the streets and got hold of the first man she could find.

A highly neurotic and hysterical, but very engaging, young lady was a patient of mine for some months for very obstinate uterine disease. She was intensely attached to a gentleman who visited her occasionally, and I have every reason to know she had often yielded to his embraces. Sometimes, however, the violent revulsion of feeling she displayed to me, when speaking of him in the most violent terms of hatred, was very remarkable. She was a woman, clearly, of very strong passions, which, at times, had the complete mastery over her. She charged her own brother with having entered her room at night and ravished her. She afterward developed suicidal mania, became very violent, was placed in an asylum, and died in a week.

These nymphomania cases manifest how completely and entirely these diseases have their origin in the nervous centers, and they are often fatal. Now I believe that, in many instances, women who bring forward false charges against medical men and others, could, if cognizant of their tendency, and willing, completely restrain themselves. But, to a certain degree, it is a pleasurable excitement, and, like an ordinary hysterical fit, if it is allowed to go beyond a certain point, then it becomes absolutely uncontrollable. The *lying* propensity then, especially, gets beyond all their powers of repression; the desire for sympathy, notoriety and pity becomes morbidly strong, and they care not any more than the most undoubted maniac if, by indulging in the gratification of their passionate excitement, they injure or destroy irrevocably a man's character.

I know of a case of a young lady who, in after-life, under the power of a strong religious conviction, confessed to me that the statements made to me and others over a series of years, and the symptoms so terrible which she told us she was suffering from, were not only and often greatly exaggerated, but palpable falsehoods. When she had reached a certain point, then she could no longer restrain herself. Her powers of mendacity knew no bounds. Indeed, had she been devil-possessed, she could not have spoken then more falsely.

If these data are correct, how are medical men to comport themselves when investigating the diseases of these women?

First, whenever anything like a suspicion exists in our mind that we are dealing with such a woman—and I think that we can very often get this from the surroundings—then we are bound to exercise the utmost caution and prudence.

Secondly, the examination the first time should be as brief as possible, especially any local one of the sexual organs. If again required, we should absolutely refuse to examine except in the presence or in the neighborhood of a third person, who may be called in at a moment's notice. I once adopted this plan with a certain lady whom I shall call Mrs. A., whom I was led to suspect. She had confessed to having had unnatural relations with very near relatives. She had also once been an inmate of a lunatic asylum. I had treated her and cured her of a troublesome uterine affection in past years. Once she brought me a lady, whom I shall call Mrs. B., to treat also for uterine disease. I did so, and she got well. One day, Mrs. B. informed me that Mrs. A. had charged her with criminal connection with myself, and cautioned me to beware lest she should charge me of like misconduct with herself. Anything like severity or angry outbursts against such women is a great mistake. We are not dealing with thoroughly sane women. Kindness in our manner, at the same time as firmness in our demeanor, is the most prudent course. Wives of Potiphar exist also in the present day even among hysterical women.

So far, as regards our own personal relations with these women; but as medical jurists we must insist upon a full and complete examination of the woman herself, historically, physiologically and locally, and this examination should be made in consultation with a medical expert on the side of the defendants. It is not enough to see if she has been outraged or not; the signs of rape should be certainly unequivocal. But is she addicted to masturbation? Has her mind been deteriorated by prurient ideas? These peculiarities are seldom, if ever, noted. Juries and judges are completely ignorant of the tendencies of such women, and yet these tendencies are the keys to the whole situation.

As medical jurists, we should never forget the abominable powers of mendacity of these women. Except upon the strongest corroborative evidence, the presumption is they are *liars*, plausible *liars*, cunning *liars*—liars not necessary from ill-will or even free will, but from disease. This peculiarity is, perhaps, less understood by judges and juries, and even counsel, than the prurient habitudes of these women.

I trust I may be excused for having dwelt at such length upon so painful a subject. But it is because it is so painful, and fraught with so much danger to each one of us, that it should be fully discussed.—*Medical Press, London—Medical Abstract, N. Y.*

